# Impaired glucose and lipid metabolism in ageing aryl hydrocarbon receptor deficient mice

**DOI:** 10.17179/excli2015-638

**Published:** 2015-11-18

**Authors:** Daniel Biljes, Christiane Hammerschmidt-Kamper, Stephanie Kadow, Patrick Diel, Carmen Weigt, Volker Burkart, Charlotte Esser

**Affiliations:** 1 Leibniz-Research Institute for Environmental Medicine, Auf´m Hennekamp 50, 40225 Düsseldorf, Germany; 2 University of Essen, Institute for Molecular Biology, Hufelandstr. 55, 45147 Essen, Germany; 3 Deutsche Sporthochschule Köln, Institut für Kreislaufforschung und Sportmedizin, Am Sportpark Müngersdorf 6, 50933 Köln, Germany; 4 Institute for Clinical Diabetology, German Diabetes Center, Leibniz Center for Diabetes Research, Auf´m Hennekamp 65, 40225 Düsseldorf, Germany; 5 German Center for Diabetes Research (DZD), 85764 München-Neuherberg, Germany

**Keywords:** AHR, metabolic syndrome, diet, dyslipidemia, senescence

## Abstract

Disturbed homeostasis of glucose and lipid metabolism are dominant features of the so-called metabolic syndrome (MetS) and can increase the risk for the development of type 2 diabetes (T2D), a severe metabolic disease. T2D prevalence increases with age. The aryl hydrocarbon receptor (AHR) is a sensor of small molecules including dietary components. AHR has been identified as potential regulator of glucose homeostasis and lipid metabolism. Epidemiologically, exposure to xenobiotic AHR ligands such as polycyclic aromatic hydrocarbons is linked to T2D. We assess here the potential role of the AHR in disturbances of glucose and lipid metabolism in young (age 2-5 months) and old (age > 1,5 years) AHR-deficient (AHR KO) mice. Fasted young wildtype (WT) and AHR-KO mice displayed similar blood glucose kinetics after challenge with intra-peritoneal glucose injection. However, old AHR-KO mice showed lower tolerance than WT to i.p. administered glucose, i.e. glucose levels rose higher and returned more slowly to normal levels. Old mice had overall higher insulin levels than young mice, and old AHR-KO had a somewhat disturbed insulin kinetic in the serum after glucose challenge. Surprisingly, young AHR-KO mice had significantly lower triglycerides, cholesterol, high density lipoprotein values than WT, i.e., a dyslipidemic profile. With ageing, AHR-KO and WT mice did not differ in these lipid levels, except for slightly reduced levels of triglycerides and cholesterol. In conclusion, our findings in AHR KO mice suggest that AHR expression is relevant for the maintenance of glucose and lipid homeostasis in old mice.

## Abbreviation

Aryl hydrocarbon receptor (AHR); aryl hydrocarbon receptor deficient mouse (AHR-KO); aryl hydrocarbon receptor nuclear transporter (ARNT); dioxin-responsive element (DRE); glucose tolerance test (GTT); high density lipoprotein (HDL); low density lipoprotein (LDL); metabolic syndrome (MetS); polycyclic aromatic hydrocarbons (PAH); type 2 diabetes (T2D); triglyceride (TG); very low density lipoprotein (VLDL); wildtype mouse (WT)

## Introduction

Diabetes mellitus is characterized by high blood glucose levels, caused either by lack of insulin due to autoimmune destruction of the insulin secreting cells in the pancreas (Type 1 diabetes), or by reduced insulin sensitivity of insulin responsive tissues, so-called insulin-resistance (Type 2 diabetes, T2D) or by a combination of both disorders (ADA, 2014[1]) In either condition, regulation of glucose homeostasis is impaired. Long term consequences of untreated T2D are cardiovascular damage, kidney complications, blindness, and impaired wound healing. T2D, by far the most frequent form of diabetes affects more than 300 Million people worldwide, develops with age, and is often associated with changes in glucose and lipid metabolism, obesity, and/or hypertension. Detection of some or all of these parameters is frequently referred to as the “metabolic syndrome”, and by some considered a warning sign of developing T2D (Prasad et al., 2012[30]). The factors which can contribute to the broad inter-individual variance and contribution of these changes in metabolic parameters are not clear. However, age is a major risk factor for the development of disturbances of glucose and lipid metabolism. 

Recent epidemiological studies have revealed a link between environmental factors, especially traffic-related air pollution, and diabetes or metabolic syndrome (Hutcheson and Rocic, 2012[14]; Kramer et al., 2010[18]; Rajagopalan and Brook, 2012[31]). Moreover, persistent organic pollutants and polycyclic aromatic hydrocarbons (PAH), notably 2,3,7,8-tetrachlorodibeno-*p*-dioxin (TCDD), are associated with insulin resistance and T2D. A significant increase in T2D incidence was observed in 2,3,7,8-TCDD-exposed indivi-duals in the Yusho accident in Japan, in Vietnam Veterans, and in the victims of the Seveso-incident 1976 in Italy (Bertazzi et al., 2001[2]; Kashima et al., 2015[15]; Warner et al., 2013[40]). The mechanisms are not clear, but possibly inflammatory cytokines such as TNFα contribute to a lower availability of the glucose transporter Glut4 in muscle cells. (Fujiyoshi et al., 2006[11]).

The transcription factor AHR, a member of an evolutionarily old protein family, is a sensor of small chemicals, including natural dietary substances, but also man-made PAHs such as TCDD and other environmental pollutants. Binding of AHR to these chemicals (called “ligands”) triggers adaptive and cell-specific intracellular responses. AHR resides in the cytoplasm complexed with accessory molecules (hsp90 and others). Binding of ligands changes the conformation of AHR and exposes an importin binding site. AHR moves into the nucleus where it sheds the accessory proteins and binds to partner molecule ARNT; together they form a functional transcription factor. AHR/ARNT binds to short promoter elements called “dioxin responsive elements”, DRE. Numerous genes have DRE sites in their promoters. Historically, AHR was first recognized as the ma-ster regulator of a battery of xenobiotic metabolizing enzymes, such as CYP4501A1. More recent research demonstrated its involvement in the modulation of stress genes and genes of gluconeogenesis and lipid metabolism (Dere et al., 2011[8]; Diani-Moore et al., 2010[9]; Minami et al., 2008[22]). AHR is an important signaling molecule for immune responses, notably for the balance between inflammatory and regulatory T cell response, and for differentiation programs of innate immune cells (Kiss et al., 2011;[17] Lee et al., 2011[20]; Nguyen et al., 2013[24]; Stockinger et al., 2014[34]). Both persistent and strong activation by non-degradable molecules such as TCDD and complete lack of AHR impairs important functions (Esser C, 2014[10]). By now, it is recognized that there is a healthy level for AHR activated by physiological ligands. 

Exposure to the high affinity AHR ligand TCDD has long been known to decrease serum glucose and triglyceride concentrations, and to cause progressive weight loss and deadly cachexia at high doses (30-day LD_50_ for C57BL/6 mice 182 µg/kg body weight) (Chapman and Schiller, 1985[5]; Poland and Knutson, 1982[29]). More recently, genome wide-expression analyses identified gene networks involved in lipid metabolism as changed by TCDD exposure in mice and rats. A single oral dose of 30 µg/kg TCDD resulted in gene responses involving steroid, lipid and carbohydrate metabolism in hepatic tissue of mice (Nault et al., 2013[23]). In murine adipose tissue, expression of inflammatory genes dominated upon TCDD treatment compared to controls (Kim et al., 2012[16]). Moreover, epidemiological research suggests that exposure to pollution containing potential AHR-ligands contribute to inflammation, obesity and cardiovascular disease (Consonni et al., 2008[6]; Cranmer et al., 2000[7]; Kramer et al., 2010[18]). It remains unclear to what extent the systemic lack of AHR - as opposed to its activation by TCDD - affects lipid and glucose metabolism, or parameters of metabolic syndrome, especially when animals age. To address this gap, we analyzed key parameters of glucose and lipid metabolism in young and old AHR-deficient mice.

## Material and Methods

### Mice

AHR-deficient mice (Schmidt et al., 1996[33]) and C57BL/6 wild-type mice, or littermates as controls were used. Mice received standard chow and water *ad libitum*, and lived on a 12/12 light-dark cycle. Experiments were done with permission, in accordance with relevant German animal welfare laws. 

### Measurement of glucose and glucose tolerance 

For glucose tolerance tests (GTT), mice were fasted for 15 hours. Mice were injected i.p. with a 20 % glucose solution in saline giving 2 g/kg body weight. Blood glucose was measured at 0, 15, 30, 60, 90, and 120 min from a drop of tail vein blood, using l Freestyle lite glucometer (Abbot, Alameda, CA, USA). 

### Determination of serum insulin levels

Insulin was measured from sera using a solid phase two-site enzyme immunoassay based on the sandwich technique (Ultrasensitive Mouse Insulin ELISA; Mercodia, Uppsala, Sweden). In brief, 5 µl of the serum samples were added to each well of a 96 well plate pre-coated with an antibody against a distinct antigenic determinant of the insulin molecule. A peroxidase-conjugated antibody directed against a separate insulin epitope was added. After 6 h of incubation at room temperature, TMB as substrate was added for 15 min. Thereafter, stop solution was added, the O.D. of the resulting solution was measured in a multichannel photometer at 450 nm, and the insulin concentrations were quantified from a standard curve. 

### TG, HDL, LDL and cholesterin measurements 

Serum was obtained by centrifugation at 4 °C and 3000 x g and stored at -20 °C. Serum levels of triglyceride were analyzed by colorimetry using ABX Pentra reagent (ABX Diagnostics Montpellier, France). The determination of cholesterol, HDL and LDL was done by photometry using reagents from DIALAB (Wiener Neudorf, Austria). To measure the serum lipids a chemistry analyzer (Roche Hitachi Cobas Mira Plus) was used. Concentrations of VLDL were calculated by subtraction of the subunits LDL and HDL from total cholesterol.

### Statistical analysis

Data were analyzed with GraphPad Prism^®^ software. Data are expressed as mean ± SD and analyzed using unpaired two tailed Student t test. A significant difference was considered when P < 0.05.

## Results

### Oral glucose tolerance is impaired in AHR-KO mice

**Figure 1:**
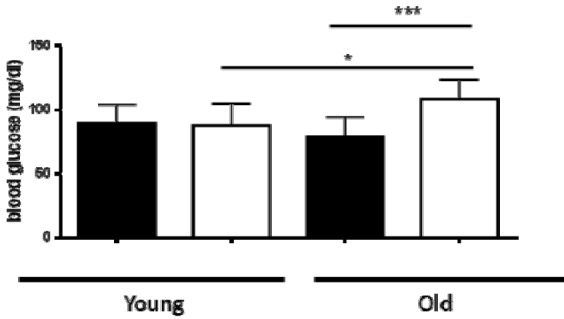
Glucose levels in fasted young and old WT and AHR-KO mice WT (black bars) and AHR-KO (white bars) mice were fasted for 15 hours and then their blood glucose levels were measured. Mice were < 4 months (young) or > 15 months (old) of age. Student´s t-test *** P < 0.001; * P < 0.05. N = 4 (young mice) or 10-12 (old mice). Means +/- SD

We tested whether there is a difference in glucose homeostasis and glucose tolerance in young (< 5 months) versus old mice (> 18 months). We fasted mice over-night and measured their blood glucose the following morning. As shown in Figure 1(Fig. 1), in young mice there was no significant difference between the WT and AHR-KO mice. The mean fasted blood glucose level was 89.5 ± 14.6 mg/dl for WT and 87.0 ± 18.6 mg/dl for AHR-KO mice, respectively. For young mice, which had not been fasted, the glucose levels were generally higher (141.3 ± 16.6 in WT vs. 129.3 ± 9.0 mg/dl in AHR-KO), but also not significantly different (data not shown). However, we observed a significant difference in old mice. AHR-KO mice had mean fasted blood glucose values of 108.2 ± 15.6 mg/dl and WT mice 78.7 ± 15.4 mg /dl. In other words, old AHR-KO mice had appr. 25 % higher glucose levels than old WT. Indeed, in old mice which had not been fasted over-night, the mean glucose level difference between AHR-KO and WT was even higher (156.6 ± 18.3 vs. 109.2 ± 12.1 mg/dl, data not shown). 

Interestingly, in fasted WT mice the blood glucose levels did not change with age, whereas in fasted AHR-KO mice the blood glucose levels were significantly higher in old than in young animals (p = 0.039). Also for mice, which had not been fasted, only the AHR-KO mice, but not the WT mice, had higher blood glucose levels in old versus young mice (data not shown). Taken together, the results demonstrate that old AHR-KO mice have higher blood glucose levels in comparison to old WT mice, and AHR-KO cannot maintain blood glucose homeostasis with age. 

**Figure 2:**
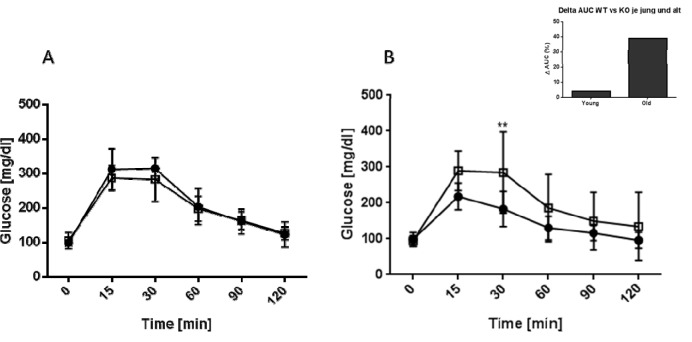
Kinetics of blood glucose levels in young and old mice after GTT C57BL/6 wildtype (black squares) and AHR-KO mice (white squares) were injected i.p with 2 g glucose/kg body weight and their blood glucose levels monitored for two hours at the indicated time points. (A) young mice (2-4 months), (B) old mice (18-24 months). Insert: Percent difference between the areas under the curve of WT versus AHR-KO for the young and old mice. Data shown are of two pooled independent experiments, N = 8 for young mice, N = 9 for old mice, each genotype. *P < 0.05; **P < 0.001

To further assess the ability of AHR KO mice to maintain glucose homeostasis we performed a glucose tolerance test. Mice were injected with glucose i.p. and their blood glucose levels measured for up to two hours. The results for young and old mice are shown in Figure (Fig. 2)2. Both strains and age groups were capable of down-regulating blood glucose levels to normal values within two hours. However, old AHR-KO mice displayed significantly greater difficulties in coping with the glucose challenge, i.e. their blood glucose concentrations initially increased to higher levels, then returned more slowly to normal values than in WT mice. When calculated as area under the cure (AUC), values were 39 % higher in old AHR-KO than in old WT (Figure 2, Insert(Fig. 2)). 

### Insulin kinetics in WT and AHR-KO in glucose challenged mice

**Figure 3:**
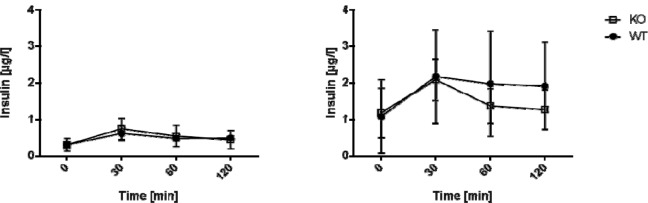
Insulin levels in old mice after GTT C57BL/6 wildtype and AHR-KO mice were injected i.p. with 2 g glucose/kg body weight and insulin levels were monitored for up to two hours at the indicated time points. AHR-KO (white squares), WT (black circles). Differences in insulin levels between WT and AHR-KO are not statistically significant. (A) young mice, (B) old mice.

**Figure 4:**
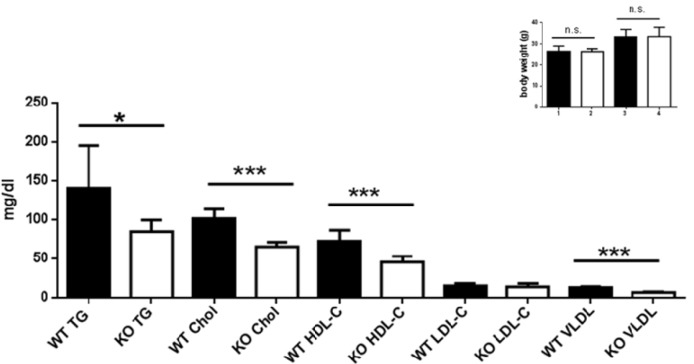
Lipid profile of young WT and AHR-KO Serum lipid measurements of postprandial young WT and AHR-KO mice. TG: Triglycerides; Chol: cholesterol; HDL-C: High density lipoprotein; LDL-C: low density lipoprotein. N = 10 (WT, black bars) and 10 (AHR-KO, white bars), partly pooled for measurement. *P < 0.05, ***P < 0.0001; Insert: Body weight of young (1,2) and old (3,4) WT (black bars) or AHR-KO (white bars).

To test whether the impaired glucose tolerance in AHR KO mice is caused by reduced insulin release we assessed the insulin secretory capacity of the animals. Insulin levels were determined during a glucose tolerance test (GTT) in WT versus AHR-KO mice (Figure 3(Fig. 3)). In young mice, we detected no difference between the insulin kinetics after glucose challenge (Figure 3A(Fig. 3)). In old mice, basal insulin levels were higher on average than in young mice, and insulin levels differed strongly between individuals. Insulin levels increased in response to the glucose challenge in both WT and AHR-KO lines, on average two-fold. Levels returned to basal values within the observation period. Histologically, there was no apparent difference in size and number of islets in the pancreas in either strain (data not shown). We did not observe different body weights between AHR-KO and WT mice (see Figure 4, Insert(Fig. 4)). The average weight was 26.5 ± 2.5 g and 26.4 ± 1.5 for three months old WT and AHR-KO, and 33.4 ± 3.9 and 33.7 ± 4.0 for two years old WT and AHR-KO, respectively (N = 30 and 19). Similarly, there was no difference in the wet weight of epididymal fat tissue in young mice (data not shown).

In aggregate, AHR-KO mice have an impaired capacity to quickly regulate blood glucose levels despite the ability to upregulate insulin release upon glucose challenge. This worsens with age and eventually old AHR-KO mice have higher blood glucose levels than WT mice. 

### Dyslipidemia in old and young WT and AHR-KO mice 

Besides dysregulated glucose homeostasis, derangements of lipid metabolism, particularly increased triglycerides and LDL are major diabetes-promoting metabolic disorders. We therefore measured TG, cholesterol, HDL-C, LDL-C and VLDL in the serum of young AHR-KO and WT mice, and of 18-21 months old AHR-KO mice. As shown in Figure 4(Fig. 4) and Table 1(Tab. 1), we found that TG, cholesterol, HDL-C, and VLDL values were significantly lower in young AHR-KO mice compared to age-matched WT animals. LDL levels did not differ between the two genotypes. There was no difference in any of these lipid marker between old and young mice; thus, there was no age associated change in the overall serum lipid markers. For TG this has been reported before (Traber et al., 2010[38]). Aged AHR-KO were an exception, as they had slightly more cholesterol. The significantly lower values for cholesterol in young AHR-KO vs. young WT leveled out with age. Overall, values for young WT mice are similar to those reported in the literature and relevant databases (see http://www.phenome.jax.org). As expected, we found that fasted mice had lower values for TG. When we stratified data of the old mice for ´fasted versus post-prandial´, only LDL-C was different between fasted WT and AHR-KO (higher), all the other markers were similar between genotypes. AHR-KO mice were more resistant to post-prandial TG increase, and prone to post-prandial HDL-C decrease. Mice gained weight with age, but there was no difference between genotypes (see Figure 4: HDL, Insert(Fig. 4)). Also, epididymal adipose tissue weight did not differ between AHR-KO and WT mice at 6 months of age (data not shown).

## Discussion

T2D is a major global concern because of the sheer number of people affected, the related severe health issues, and the high costs placed on health care systems. Notably, T2D develops with age, reflecting the involvement of both environmental factors and an adverse life-style. In addition individual genetic factors might be relevant. Epidemiolo-gical and laboratory studies have provided evidence that exposure to AHR agonists, in particular polycyclic aromatic hydrocarbons such as 2,3,7,8- TCDD, affects glucose homeostasis and increases the incidence of T2D (Bock, 1994[3]; Goodman et al., 2015[12]; Warner et al., 2013[40]). While early research on AHR has focused on toxic activation by polycyclic aromatic hydrocarbons, i.e., persistent environmental pollutants, recent studies have highlighted important roles for AHR in normal development and physiology (Pascussi et al., 2008[26]; Swedenborg et al., 2009[36]). It has long been known that AHR signaling can affect metabolism, including of glucose and lipids, and mitochondria function (Park et al., 2013[25]). Nonetheless, few studies have looked at glucose homeostasis in AHR-deficient mice, and none explicitly looked at older mice. We here report dyslipidemia and dysbalanced glucose homeostasis in AHR-KO mice. Both parameters are known to contribute to the development of T2D in humans.

We detected an impaired glucose tolerance in old AHR-KO mice. An independent study of Thackaberry et al. (2003[37]) reported no difference in oral glucose tolerance (GT) in pregnant mice, but a somewhat higher oral GT in non-pregnant young female mice, and overt GT in 23 % of seven months old female AHR-deficient mice. The authors suggested that glucose intolerance is an age effect. Our data on much older mice confirm and extend this idea. The Thackaberry group reported decreased serum insulin levels in their seven months old female mice. In another study, the group of S. Tischkau reported improved oral GT in young mice (mixed male and female) (Wang et al., 2011[39]). We currently have no explanation for these contrasting results, but note that basal fasting glucose was higher as well in their study. It is unclear whether this is due to a sex-specific effect or the much higher age in our study. It will be important to analyze further possibly influencing factors such as sex, stress, housing conditions, specific chow, or the inadvertent possibility of genetic drift in mouse lines of various laboratories (Phillips et al., 1999[28]). 

Lipids are transported in blood complexed with many proteins, which collectively are called HDL or LDL. Cholesterol and TG are complexed within LDL, and high levels of LDL are risk factors for atherosclerosis and cardiovascular disease. The mouse lipoproteom resembles closely the human situation, and the mouse is a relevant model to study questions associated with, e.g., HDL levels (Gordon et al., 2015[13]). Increased TG and LDL, together with lower HDL establish an “atherogenic lipid profile”, which may be associated with cardiovascular risk. As shown in Figure 4(Fig. 4) for young mice, we found that TG levels in postprandial AHR-KO, rather than being increased, were significantly lower than in wildtype. However, consistent with an atherogenic profile and symptoms of the so-called metabolic syndrome, HDL levels were significantly lower in AHR-KO. LDL did not differ between the two genotypes. VLDL was significantly lower in serum of AHR-KO mice than in wildtype littermates. Unexpectedly, there was no difference in any of these lipid markers between old and young AHR-KO, thus there was no age associated change in the AHR-KO mice with respect to these parameters. The expression of the enzyme LpL (lipoprotein lipase) increases with age in the livers of both WT and AHR-KO mice, and was reportedly higher in 60-week old AHR-KO than WT mice (Minami et al., 2008[22]). Lpl is the enzyme which dissolves lipoproteins and thus can release TG. It is known that AHR activation can induce steatosis, and an accumulation of TG (Lee et al., 2010[19]). Moreover, Lpl has a putative DRE in its promoter (Sun et al., 2004[35]) and might thus need AHR for constitutive expression; together this might explain the low TG levels in AHR-KO mice. Curiously, we did not find obesity associated with AHR-deficiency in mice. This is congruent with data from other groups and a second AHR-KO strain as well (Sato et al., 2008[32]; Xu et al., 2015[42]). Physical activity is a major factor in developing metabolic syndrome. A recent study suggested that a low affinity AHR allele leads to higher activity (Williams et al., 2014[41]). Indeed, considering that AHR is part of a gene-network in humans responsive to physical activity, such an influence is possible (Phillips et al., 2013[27]). Moreover, recent data shows that AHR is involved in energy expenditure in experimentally induced obesity as well (Xu et al., 2015[42]). Thus a highly complex picture emerges, where AHR has positive impact in some ways, but is not beneficial in other metabolism related aspects. 

Our data suggest that AHR-deficiency will in the long run be detrimental to metabolism. AHR-mediated gene regulation, which has been reported for hundreds of genes (Sun et al., 2004[35]) in young mice, will conceivably differ in old mice, and more research is needed to identify geriatric gene regulation and the environmental parameters which might shape it. 

Removal of AHR ligands from the diet of mice can result in similar symptoms as genetic AHR deficiency. This has been shown in studies looking at gut barrier, immune cell development and infection susceptibility (Kiss et al., 2011[17]; Li et al., 2011[21]). It has therefore been suggested, that the diet will have major influences on “healthy” AHR signaling, which needs to be not too low, and not too high/persistent (as evident from the data with TCDD and other xenobiotic AHR-ligands). A host of epidemiological studies has cemented the beneficial effects of certain diets, especially diets rich in vegetables (Calton et al., 2014[4]). Our data are very well congruent with this, and suggest a causal molecular mechanism, which was previously overlooked. 

In conclusion, or data highlight involvement of AHR in glucose and lipid metabolism. As AHR-ligand withdrawal in the diet can mimic the outcome of AHR-deficiency in mice, proper consideration must be given to the potential risk of chronic AHR-ligand low (i.e. too few vegetables) diets which do not support the AHR signaling system in a physiological way.

## Acknowledgements

We thank Babette Martiensen and Waltraud Fingberg for expert technical help. 

The work of C.E. is supported by grants from the Deutsche Forschungsgemeinschaft (ES103/6-1).

## Figures and Tables

**Table 1:**
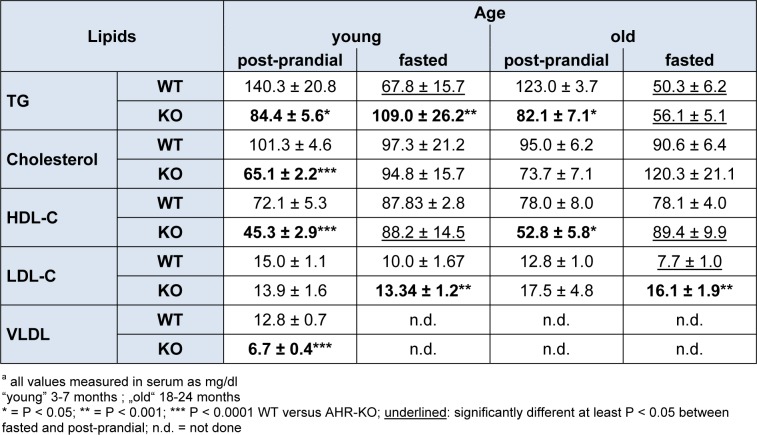
Lipids in serum^a^
